# Supplementary figures and images for: Blood RNA signatures predict recent tuberculosis exposure in mice, macaques and humans
Source: Sci Rep. 2020 Oct 9;10:16873. doi: 10.1038/s41598-020-73942-z (PMC7547102; doi:10.1038/s41598-020-73942-z)

# Classification of Time Period Post Infection

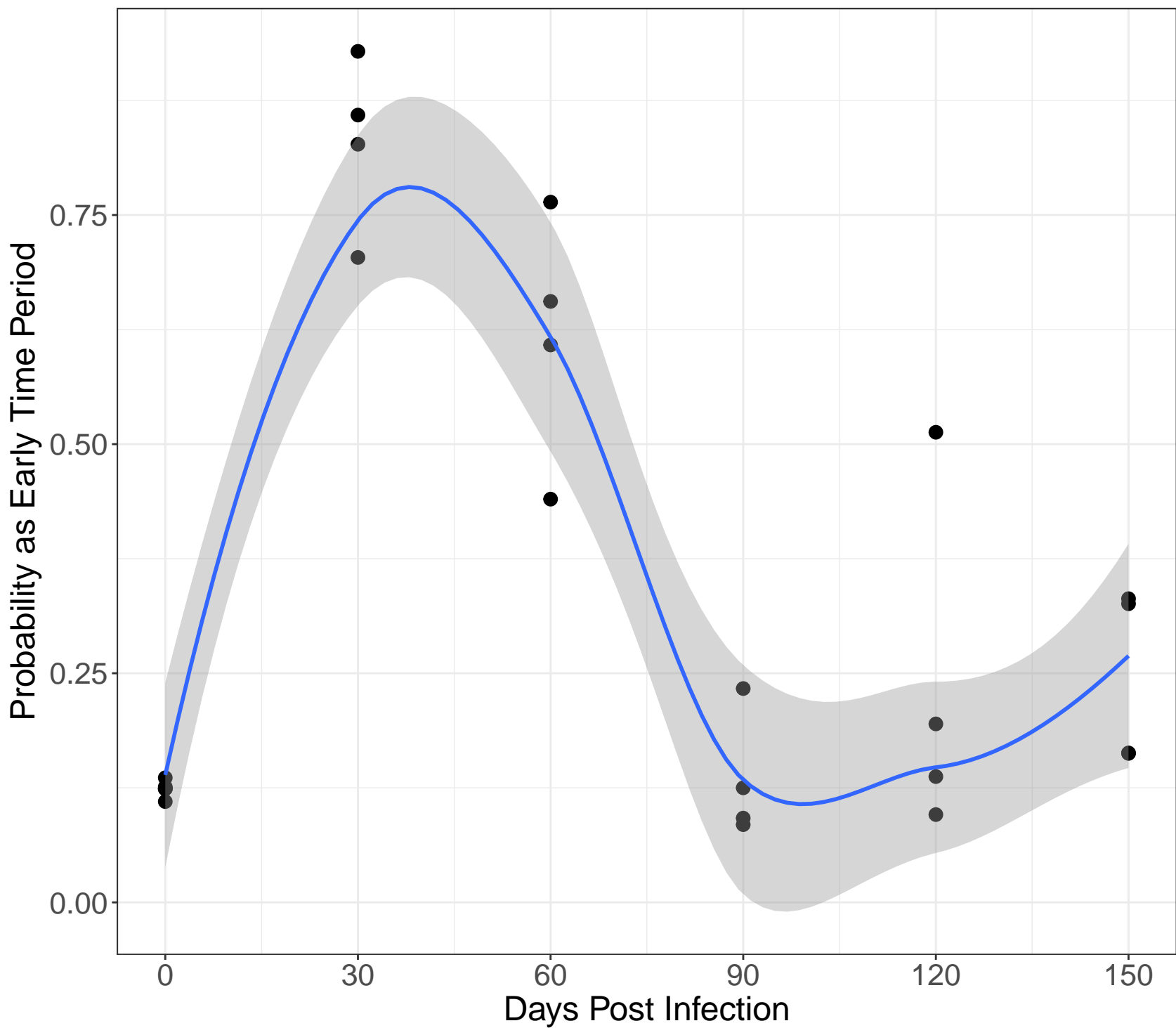

Supplement: Supplementary file 1 — Supplementary Figure S1. [file 41598_2020_73942_MOESM1_ESM.pdf]

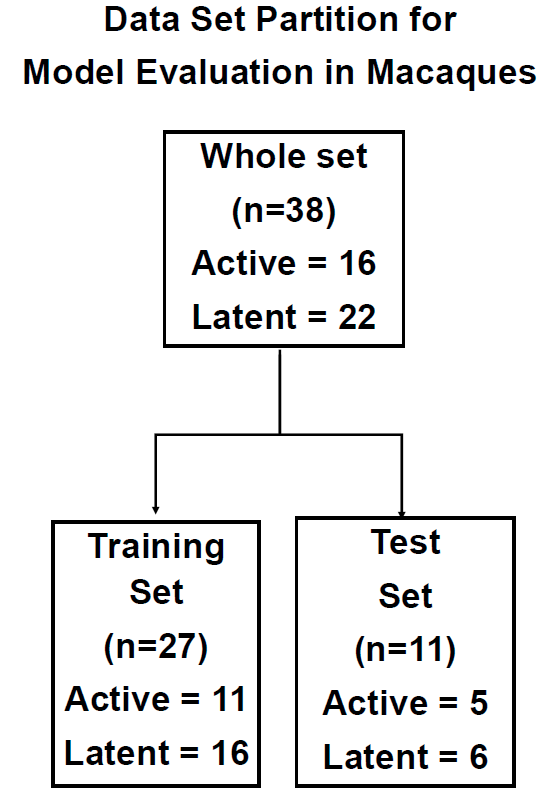

Supplement: Supplementary file 2 — Supplementary Figure S2. [file 41598_2020_73942_MOESM2_ESM.tiff]

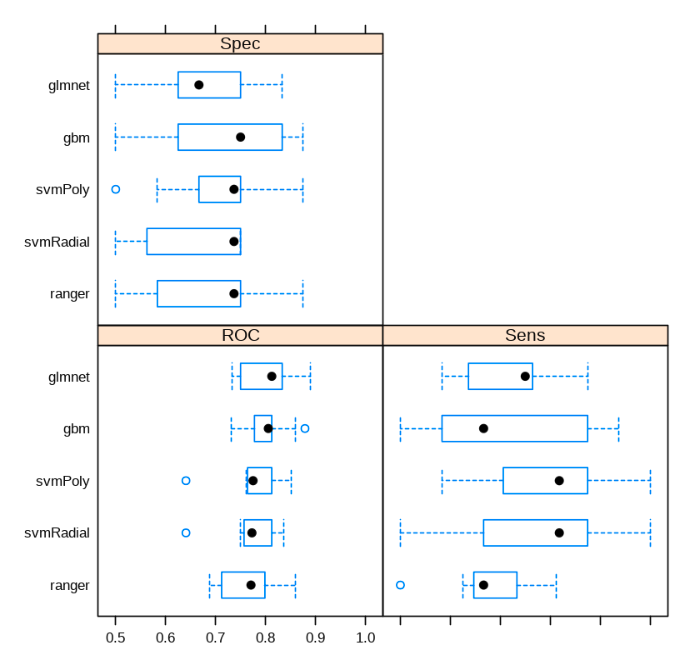

Supplement: Supplementary file 3 — Supplementary Figure S3. [file 41598_2020_73942_MOESM3_ESM.tiff]

# Classification of Time Period Post Infection

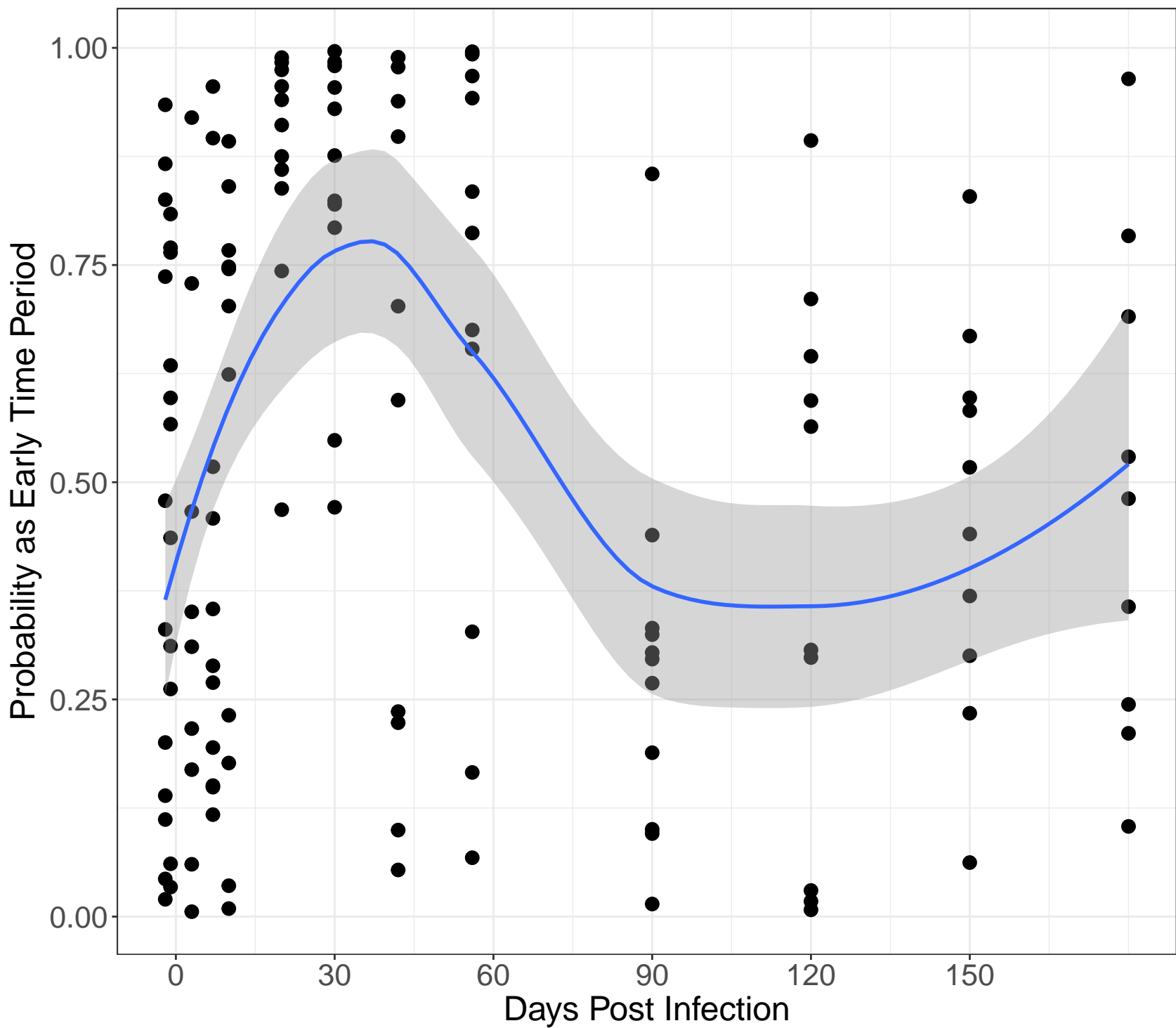

Supplement: Supplementary file 4 — Supplementary Figure S4. [file 41598_2020_73942_MOESM4_ESM.pdf]

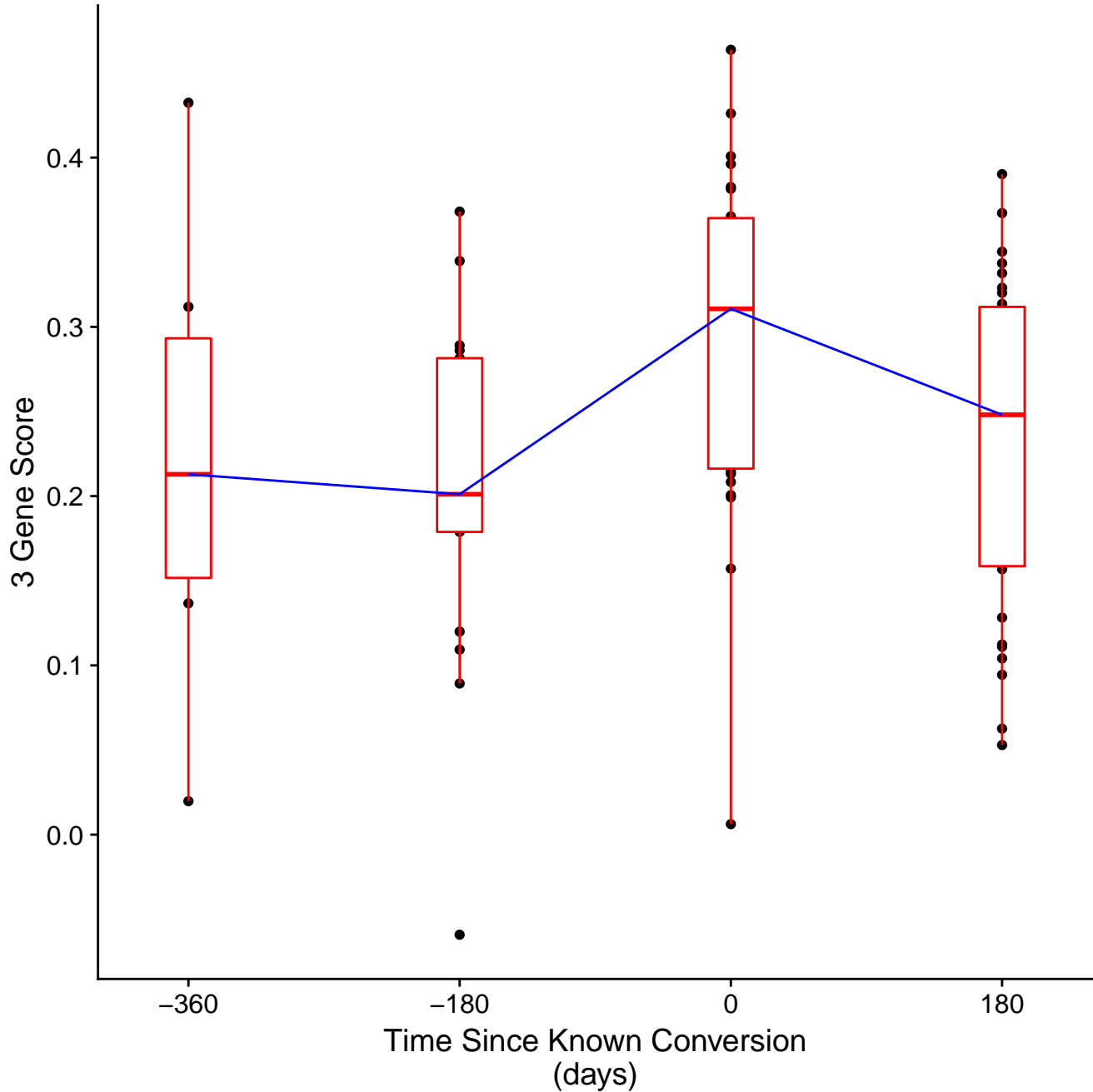

Supplement: Supplementary file 5 — Supplementary Figure S5. [file 41598_2020_73942_MOESM5_ESM.pdf]
