# Supplementary material for: Blood RNA signatures predict recent tuberculosis exposure in mice, macaques and humans
Source: Sci Rep. 2020 Oct 9;10:16873. doi: 10.1038/s41598-020-73942-z (PMC7547102; doi:10.1038/s41598-020-73942-z)
Supplement: Supplementary file 7 — Supplementary Information. [file 41598_2020_73942_MOESM7_ESM.pdf]

Figure S1. Early *vs.* late *M.tb* infection time period classification model predictions at all time points in mice. Random Forest Classifier predictions are expressed as probability of being from the early (30-60 days) time period. Predictions for *M.tb* infected mice (30-150 days; n = 20 mice) are the out-of-bag predictions of the model, whereas predictions for uninfected mice (0 days; n = 4 mice) are predictions of the model trained on all *M.tb* infected mice. Fit curve calculated via the Loess method with 95% CI shown.

Figure S2. Training and test set partition for cohort of cynomolgus macaques. Active = Developed active TB during the 6 months of study follow-up. Latent = did not develop active TB in this study. n corresponds to individual macaques, each of which underwent longitudinal sampling.

Figure S3. Comparison of different machine algorithms to predict time period of *M.tb* infection in cynomolgus macaques. Random hyperparameter search and 9-fold cross-validation of macaques were used on the training set to evaluate models to predict time period of infection from microarray data. Median (point), interquartile ranges (boxes), and ranges (whiskers) are shown for predictions on each independent fold for the best performing model for each algorithm. glmnet = Regularized Logistic Regression, gbm = Gradient Boosted Machines, svmPoly = Support Vector Machines with Polynomial kernel, svmRadial = Support Vector Machines with RBF kernel, ranger = Random Forest. Sens=Sensitivity, Spec=Specificity, ROC= Area under the curve.

Figure S4. Early *vs.* late *M.tb* infection time period classification model predictions at all time points in cynomolgus macaques. Regularized Logistic Regression predictions are expressed as probability of being from the early (20-56 days) time period. Predictions shown are final model predictions on the test set (n =

137 samples). Pre-infection samples are shown as before day 0, the day of infection. Fit curve calculated via the Loess method with 95% CI shown.

Figure S5. Trajectory of 3-gene signature for recent *M.tb* infection before and after IGRA conversion in adolescents who acquire *M.tb* infection. One sample (score = 0.47) from 360 days after known conversion is omitted but was included in analyses of Figure 6B. Boxplots represent medians with interquartile ranges, and the blue line connects medians. n = 7 -360 days, n = 17 -180 days, n = 27 0 days, n = 30 180 days.
